# Human Gb3/CD77 synthase: a glycosyltransferase at the crossroads of immunohematology, toxicology, and cancer research

**DOI:** 10.1186/s11658-024-00658-7

**Published:** 2024-11-07

**Authors:** Katarzyna Szymczak-Kulus, Marcin Czerwinski, Radoslaw Kaczmarek

**Affiliations:** Laboratory of Glycobiology, Hirszfeld Institute of Immunology and Experimental Therapy, Polish Academy of Sciences, Weigla 12, 53-114 Wroclaw, Poland

**Keywords:** glycosyltransferase, glycosphingolipid, glycoprotein, blood group, cancer, Anderson-Fabry disease, Shiga toxin, chemoresistance

## Abstract

Human Gb3/CD77 synthase (α1,4-galactosyltransferase, P1/P^k^ synthase, UDP-galactose: β-d-galactosyl-β1-R 4-α-d-galactosyltransferase, EC 2.4.1.228) forms Galα1 → 4Gal structures on glycosphingolipids and glycoproteins. These glycans are recognized by bacterial adhesins and toxins. Globotriaosylceramide (Gb3), the major product of Gb3/CD77 synthase, is a glycosphingolipid located predominantly in plasma membrane lipid rafts, where it serves as a main receptor for Shiga toxins released by enterohemorrhagic *Escherichia coli* and *Shigella dysenteriae* of serotype 1. On the other hand, accumulation of glycans formed by Gb3/CD77 synthase contributes to the symptoms of Anderson–Fabry disease caused by α-galactosidase A deficiency. Moreover, variation in Gb3/CD77 synthase expression and activity underlies the P1PK histo-blood group system. Glycosphingolipids synthesized by the enzyme are overproduced in colorectal, gastric, pancreatic, and ovarian cancer, and elevated Gb3 biosynthesis is associated with cancer cell chemo- and radioresistance. Furthermore, Gb3/CD77 synthase acts as a key glycosyltransferase modulating ovarian cancer cell plasticity. Here, we describe the role of human Gb3/CD77 synthase and its products in the P1PK histo-blood group system, Anderson–Fabry disease, and bacterial infections. Additionally, we provide an overview of emerging evidence that Gb3/CD77 synthase and its glycosphingolipid products are involved in cancer metastasis and chemoresistance.

**Graphical Abstract:**

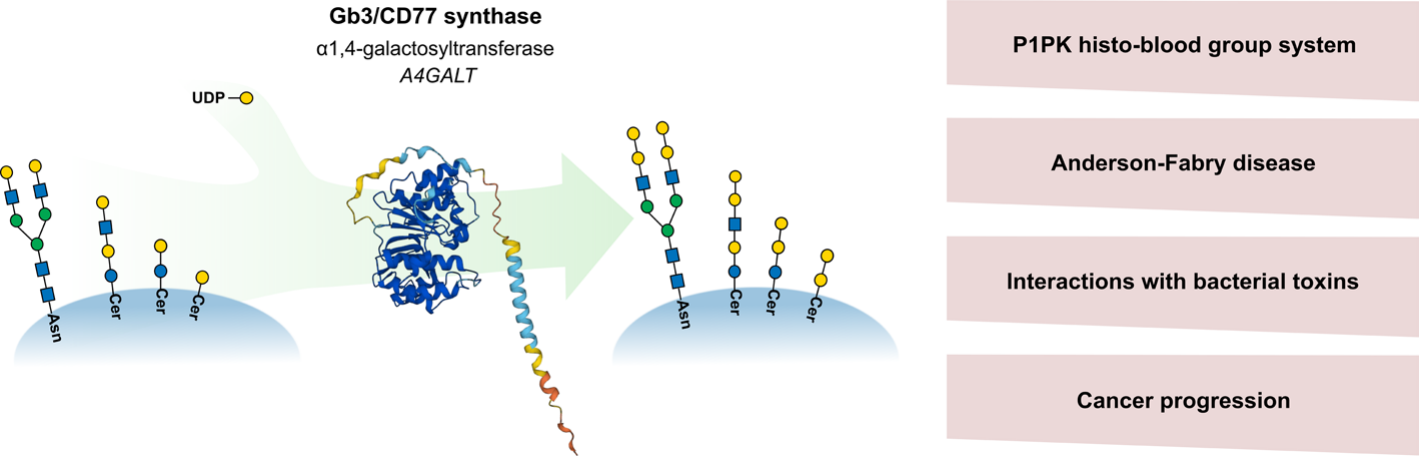

## Introduction

Human Gb3/CD77 synthase (α1,4-galactosyltransferase, UDP-galactose: β-d-galactosyl-β1-R 4-α-d-galactosyltransferase), known among immunohematologists as P1/P^k^ synthase, is encoded in humans by the *A4GALT* gene located on chromosome 22q13.2. It catalyzes the transfer of galactose residue from UDP-Gal to galactose-terminated glycosphingolipid (GSL) and glycoprotein acceptors. The many names of this enzyme reflect distinct fields in which its activity has been found relevant, such as glycobiology, immunohematology, toxicology, and oncology. Similarly, globotriaosylceramide, commonly referred to as Gb3, is also called CD77, the P^k^ antigen, or ceramide trihexoside (CTH). This may lead to misunderstandings and inaccessibility of reported data among researchers accustomed to the use of other terms.

In this review, we aim to summarize and present the most important data on the functions of Gb3/CD77 synthase and its products from different perspectives. On the one hand, glycans produced by Gb3/CD77 synthase determine phenotypes of the P1PK blood group system. On the other hand, we aim to emphasize that they are histo-blood group antigens present on many cell types other than erythrocytes, such as lymphocytes, renal endo- and epithelium, epithelium of gastrointestinal tract, or neurons. Hence, the role of these glycans as receptors for toxins and lectins released by such deadly pathogens as Shiga toxin-producing *Escherichia coli* (STEC), *Staphylococcus aureus*, or *Pseudomonas aeruginosa* is of particular significance. Moreover, since the products of Gb3/CD77 synthase are overexpressed in human colorectal, gastric, pancreatic, and high-grade serous ovarian cancer, they have diagnostic and therapeutic potential.

## Structure and localization of Gb3/CD77 synthase

Classified under the number EC 2.4.1.228, human Gb3/CD77 synthase belongs to the 32nd family of glycosyltransferases (GT32; Carbohydrate-Active enZYmes database, www.cazy.org). In silico predictions place the enzyme among type II transmembrane proteins belonging to a GT-A superfamily. Interestingly, the Gb3/CD77 synthase amino acid sequence shows the closest similarity to α1,4-*N*-acetylglucosaminyltransferase, encoded by *A4GNT* (Łukasz Sobala, personal communication).

**Fig. 1:**
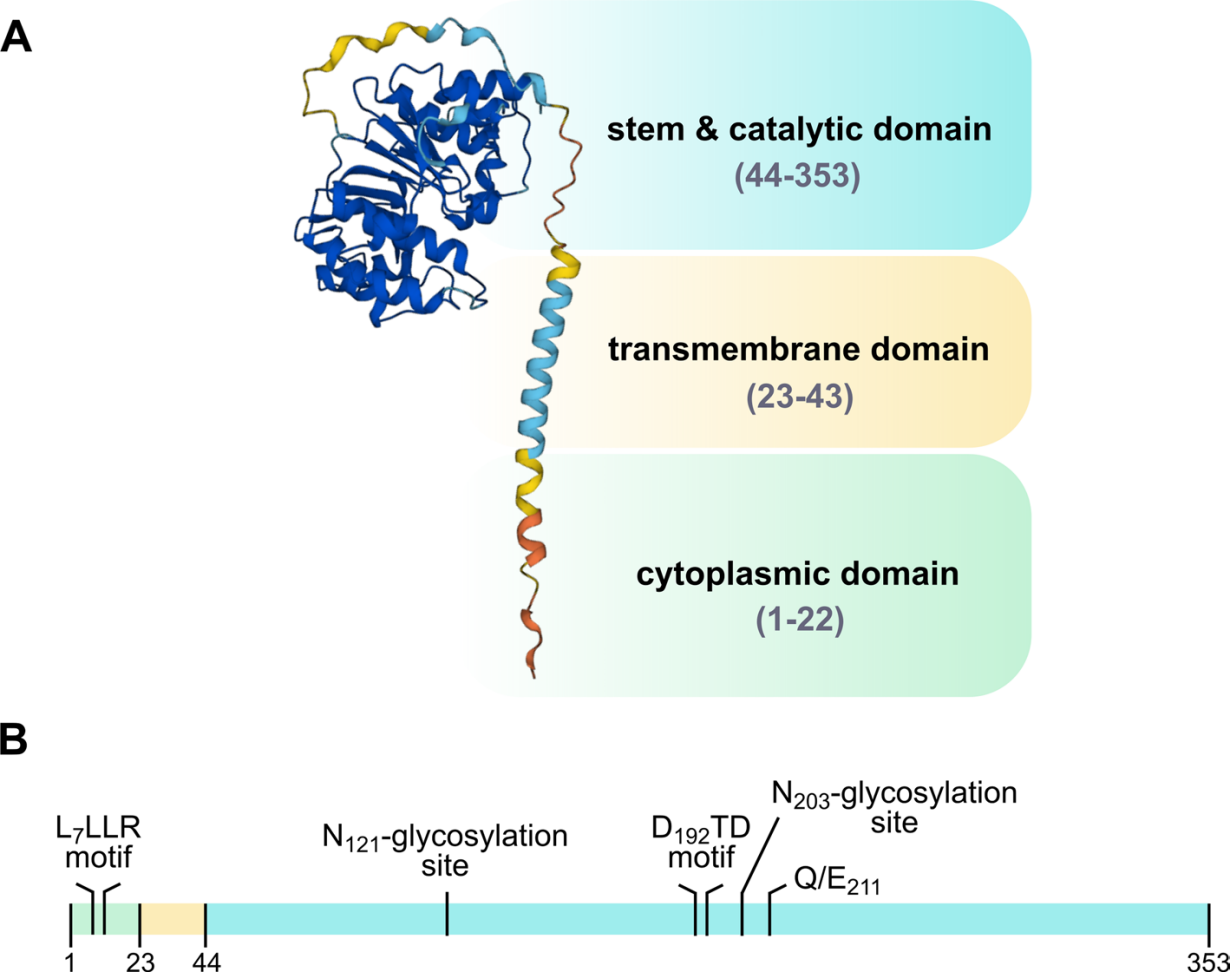
Structure of Gb3/CD77 synthase. **A** The 3D structure model predicted by AlphaFold (https://alphafold.ebi.ac.uk/entry/Q9NPC4); amino acid residues 1–22 compose the cytoplasmic tail, 23–43 form the transmembrane domain, and 44–353 encompass the stem and catalytic domain facing Golgi lumen. **B** A schematic representation of the Gb3/CD77 synthase polypeptide chain with important peptide motifs highlighted

The polypeptide chain of Gb3/CD77 synthase comprises 353 amino acids, of which residues 1–22 probably represent the cytoplasmic tail, 23–43 belong to the transmembrane domain, and 44–353 form the stem region and catalytic domain facing Golgi lumen (Fig. 1A). The catalytic domain contains a DxD motif (D_192_TD according to UniProt Q9NPC4) (Fig. 1B), which is indispensable for the enzymatic activity of Gb3/CD77 synthase, presumably through establishing interactions with divalent ions, such as Mn^2+^, at the catalytic center [1, 2]. The enzyme has two N-glycosylation sites, N_121_ and N_203_ (Fig. 1B), and both are occupied. However, while glycosylation of the N_121_ site seems dispensable, the N-glycan occupying N_203_ is crucial for Gb3/CD77 synthase localization and activity [3, 4].

Gb3/CD77 synthase resides in the *trans*-Golgi network (TGN) [3, 5, 6] where it localizes owing to the interactions with COPI vesicle-associated proteins, Golgi phosphoprotein 3 (GOLPH3) [7] and transmembrane 9 superfamily 2 protein (TM9SF2) [5, 8]. COPI-dependent retrograde transport of Golgi-resident enzymes is a crucial mechanism of the Golgi cisternal maturation model [9]. In particular, GOLPH3 and its paralog GOLPH3L control the recruitment of Golgi-resident enzymes in *trans* *cisternae* and loading into COPI-dependent retrograde vesicles [10]. Gb3/CD77 synthase is recognized by GOLPH3 through an N-terminal LxxR motif (L_7_LLR according to UniProt Q9NPC4), similarly to the lactosylceramide synthase (*B3GNT5*) and several other enzymes engaged in GSL biosynthesis [7]. Moreover, the activity of Gb3/CD77 synthase depends on interactions with lysosome-associated protein transmembrane 4α (LAPTM4A) [5, 8].

## α1,4-Galactosyltransferase activity

**Fig. 2:**
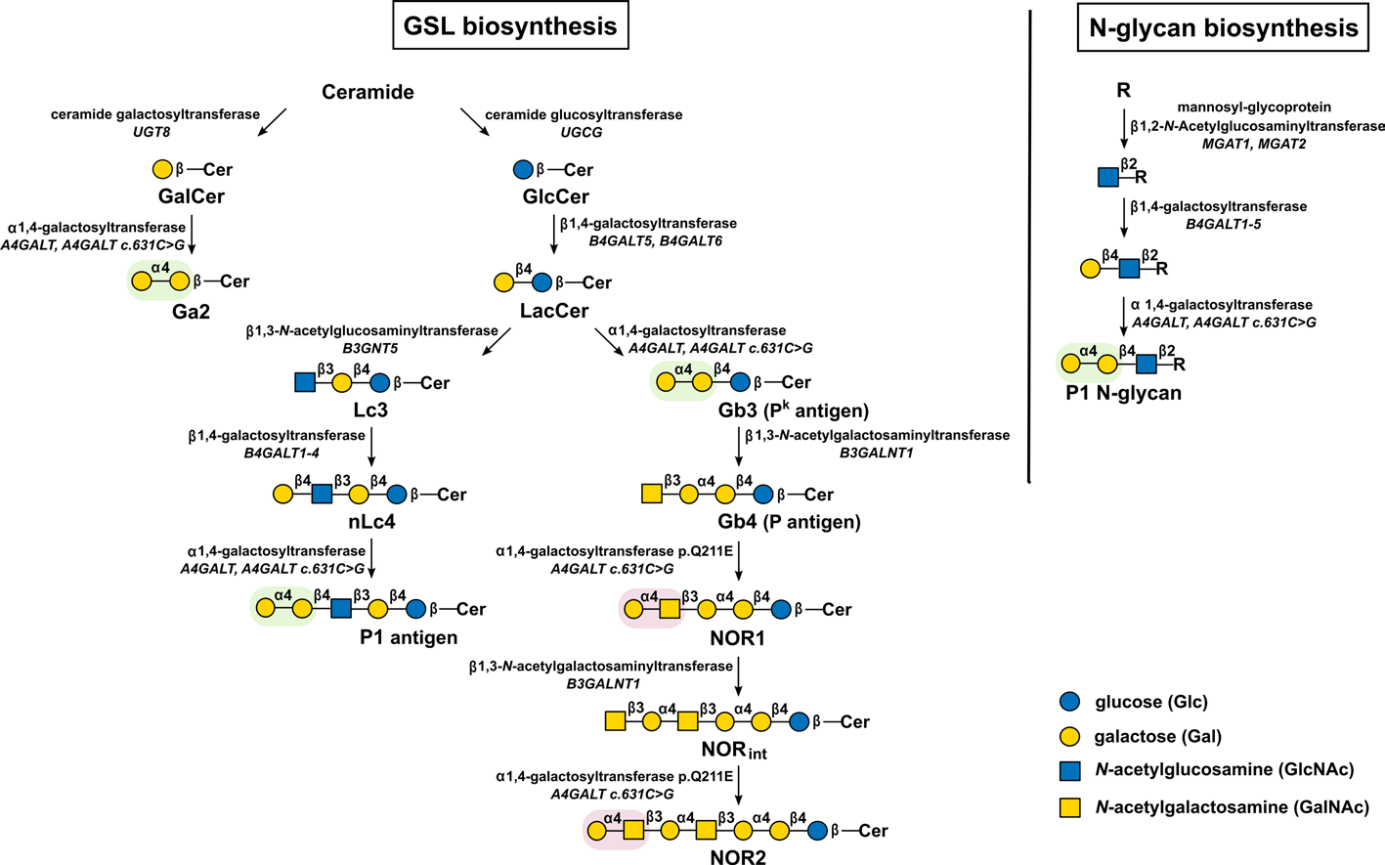
A schematic representation of the biosynthesis of GSLs and N-glycans produced by Gb3/CD77 synthase. Galα1 → 4Gal structures are highlighted in green, and Galα1 → 4GalNAc in red. Color coding according to the Symbol Nomenclature for Glycans (SNFG) [120]

Human Gb3/CD77 synthase catalyzes the transfer of galactose from UDP-Gal to galactose-terminated GSLs and N-glycan chains, creating Galα1 → 4Gal structures. The consensus human α1,4-galactosyltransferase can use several GSLs as acceptors, including galactosylceramide, lactosylceramide, and nLc4/paragloboside, giving rise to galabiosylceramide (Ga2, Gal2Cer), globotriaosylceramide (Gb3, CD77, the P^k^ antigen, CTH), and the P1 antigen (nLc5), respectively (Fig. 2) [11, 12]. Galabiosylceramide is the precursor of the gala-series, Gb3 initiates the globo-series, whereas P1 belongs to the neolacto-series of GSLs. Human Gb3/CD77 synthase was long believed to use only GSLs as acceptors. Recently, we challenged that paradigm and demonstrated that the enzyme attaches galactose residues also to N-glycans on glycoproteins [13].

Most probably, Gb3/CD77 synthase also produces P1 glycotopes (Galα1 → 4Galβ1 → 4GlcNAc) carried by long GSLs belonging to the neolacto-series: heptaosylceramide and branched decaosylceramide containing two P1 trisaccharide units. These newly identified GSL species have been recently isolated from human gastric adenocarcinoma [14].

**Fig. 3:**
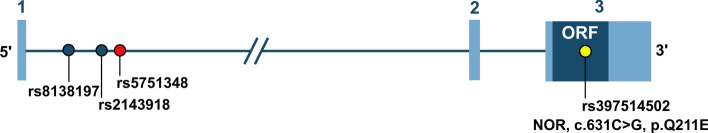
Schematic representation of the *A4GALT* gene. The rectangles depict the three exons. The circles indicate the positions of SNPs associated with P_1_/P_2_ polymorphism (rs5751348 in red, rs2143918 and rs8138197 in blue), and the yellow circle represents the rs397514502 SNP resulting in p.Q211E substitution

Astoundingly, a single point mutation (c.631C > G; rs397514502) (Fig. 3) resulting in the substitution of glutamine with glutamic acid at the 211 position of the enzyme polypeptide chain renders Gb3/CD77 synthase able to use *N*-acetylgalactosamine-terminated GSLs as acceptor substrates and synthesize the NOR antigen [12, 15]. Gb3/CD77 synthase p.Q211E (also called the NOR synthase) galactosylates globotetraosylceramide (Gb4, globoside), producing NOR1 (terminated with one Galα1 → 4GalNAc moiety [16]), which is subsequently elongated by the Gb4 synthase (β1,3-*N*-acetylgalactosaminyltransferase, *B3GALNT1*), forming NOR_int_ (Fig. 2). This GSL is further modified by the NOR synthase, leading to the formation of NOR2, encompassing two Galα1 → 4GalNAc repeats [15]. Thus, Gb3/CD77 synthase p.Q211E exhibits broadened acceptor specificity and, alongside Galα1 → 4Gal structures (on GSLs and glycoproteins), also synthesizes the Galα1 → 4GalNAc moiety of the glycosphingolipid NOR antigen. Modulation of glycosyltransferase acceptor specificity by a single amino acid substitution is unprecedented, and human Gb3/CD77 synthase remains its sole example.

The presence of NOR antigen causes the rare NOR polyagglutination syndrome [17]. The first reported case was a resident of Norton, Virginia, hence the designation “NOR” [18]. Red blood cells (RBCs) of individuals expressing NOR were agglutinated by most ABO-compatible human sera owing to the commonly occurring natural anti-NOR antibodies [18–20]. It is noteworthy that the terminal Galα1 → 4GalNAc structure of NOR is extremely unique. It was never detected before in mammals, and among vertebrates, it was reported solely in *Rana ridibunda* egg jelly coat mucin [21]. However, a similar structure (Galα1 → [4GalNAcα1 → 4Galα1]_n_ → 4GalNAc) was identified in galactosaminogalactan secreted by *Aspergillus fumigatus* [22]. Most human sera react with this polysaccharide, presumably owing to the presence of anti-NOR antibodies elicited by *A. fumigatus* infections.

## *A4GALT* gene regulation and the genetic background of the P1PK histo-blood group system

Gb3, under the name of “P^k^ antigen,” along with P1 and NOR, are the three antigens constituting the P1PK histo-blood group system (International Society of Blood Transfusion (ISBT) no. 003). The P1PK system (originally designated as “P”) was discovered by Landsteiner and Levine in 1927, and since then has undergone numerous modifications following advances in understanding of its biochemical and genetic basis (reviewed in Refs. [23, 24]). Most individuals display P_1_/P_2_ phenotypes: P_1_ when the P1 antigen along with P^k^ (Gb3) is expressed on erythrocytes, and P_2_ when only P^k^ (Gb3) is present (Table 1). P_1_/P_2_ distribution varies greatly between populations; e.g., the incidence of the P_1_ phenotype ranges from 20–30% among Asians to 75–80% in Europeans [25].

**Table 1:** The phenotypes of the P1PK histo-blood group system and *A4GALT* genotypes (ψψ does not encode any functional protein)

Phenotype	Genotype	Antigens
P_1_	*P*^*1*^*P*^*1*^	P1, P^k^
	*P*^*1*^*P*^*2*^	
P_2_	*P*^*2*^*P*^*2*^	P^k^
p	ψψ	–
P_1_NOR	*P*^*1NOR*^*P*^*1*^	P1, P^k^, NOR
	*P*^*1NOR*^*P*^*2*^	

The gene encoding Gb3/CD77 synthase (*A4GALT*) encompasses three exons, and the entire open reading frame (ORF) is contained within exon 3 (Fig. 3). Various missense, nonsense, and frameshift mutations in the *A4GALT* ORF underlie the rare p phenotype (incidence estimated at 5.8 per million), found in individuals lacking any P1PK antigens [26]. In turn, the P_1_NOR phenotype, which is characterized by the presence of P^k^ (Gb3), P1, and NOR, originates from a single point mutation c.631C > G in the *A4GALT* ORF (rs397514502) causing the p.Q211E substitution [15]. The P_1_NOR phenotype has been identified in members of only two families to date, one living in the USA and one in Poland [18, 19].

The genetic basis of the P_1_/P_2_ phenotypic polymorphism posed a long-standing conundrum because it does not arise from mutations in the *A4GALT* ORF [27]. Since individuals with the P_1_ phenotype showed increased *A4GALT* transcript levels in comparison with P_2_, and it was generally accepted that the P_1_/P_2_ polymorphism is associated with transcriptional regulation of the *A4GALT* expression [28, 29]. Throughout the years, several single-nucleotide polymorphisms (SNPs) upstream from the coding region were proposed to determine the P_1_/P_2_ status [28–30], of which rs5751348 proposed by Lai and coworkers [30] proved to be the most important [31, 32]. rs5751348 is located in the *A4GALT* intron 1 (3084 bp downstream from the transcription initiation nucleotide of exon 1, Fig. 3), near rs2143918 and rs8138197, the SNPs usually linked with rs5751348 and hence also well associated with the P_1_/P_2_ polymorphism. rs5751348G (*A4GALT P*^*1*^ allele) sits within the binding motifs for transcription factors early growth response protein 1 (EGR1) and Runt-related transcription factor 1 (RUNX1) [33, 34]. EGR1 binds specifically to the *P*^*1*^ allele, thus activating its transcription [33]. RUNX1 was shown to bind the *P*^*1*^ allele-specific probes, and its knockdown resulted in downregulation of *A4GALT* expression [34].

Some individuals with the P_2_ phenotype produce anti-P1 antibodies, but they rarely cause hemolytic transfusion reactions [35, 36]. However, individuals with the null p phenotype develop so-called anti-PP1P^k^ (formerly known as “anti-Tja”) antibodies, which recognize P^k^ (Gb3) and P1, as well as the P antigen belonging to the GLOB histo-blood group system (ISBT no. 028). The P antigen is globotetraosylceramide (Gb4), produced directly from P^k^ (Gb3) (Fig. 2) and hence also absent in p individuals. Anti-PP1P^k^ antibodies can induce severe hemolytic transfusion reactions, recurrent spontaneous abortions, and sporadically mild hemolytic disease of the fetus and newborn [37]. Interestingly, most human sera contain antibodies that can recognize a purified P^k^ antigen (Gb3), but do not bind P^k^-positive cells, nor do they agglutinate P^k^-positive erythrocytes owing to the crypticity of cell-bound P^k^ [38, 39].

## Tissue distribution of Gb3/CD77 synthase and its products

Expression of Gb3/CD77 synthase and its products is not restricted to the erythroid cell lineage. The *A4GALT* transcript is ubiquitously expressed in all human tissues, at high levels in the heart, kidney, spleen, liver, testis, and placenta and at lower levels in the lung, stomach, brain, colon, and muscle [11, 27].

Galabiosylceramide was detected in the kidney, urine, and cerebrospinal fluid [40–42]. In normal tissues, P1 antigen expression appears to be limited to erythrocytes [43]. Similarly, the P1 glycotope-capped N-glycoproteins have been found so far only on erythrocytes of *P*^*1*^*P*^*1*^ individuals [44].

However, Gb3 is widely distributed in human tissues. Its presence was reported in lymphocytes, platelets, heart, kidney, lung, liver, smooth muscle, epithelium of gastrointestinal tract, neurons, and central nervous system endothelium [43, 45–48]. Gb3 was detected in a subset of B lymphocytes in germinal centers, where it was suggested to play a role in the negative selection of B cells, hence being termed CD77 [49–52]. Recently, it was shown that Gb3 expressed on germinal center B cells promotes the translocation of CD19 to the B cell receptor complex and stimulates downstream signaling pathways [53]. Moreover, Gb3 is a major neutral GSL of T lymphocytes and macrophages [54]. In the kidney, Gb3 is expressed on endothelial as well as on epithelial tubular cells [55], where it presumably contributes to the reabsorption of low-molecular-weight proteins and albumin [56]. Moreover, Gb3 is also present in human milk and presumably may act as a decoy receptor for Shiga toxins, protecting breastfed infants from developing hemorrhagic colitis and hemolytic uremic syndrome [57, 58]. It should be noted that, in methods based on immunodetection on the cell surface, Gb3 is poorly accessible for monoclonal antibodies without protease pretreatment of the cells and hence its expression may be underestimated.

## Gb3/CD77 synthase products as culprits of Anderson–Fabry disease

Under homeostatic conditions, the products of Gb3/CD77 synthase are degraded in lysosomes by α-galactosidase A (EC 3.2.1.22), encoded by the *GLA* gene located on chromosome Xq22.1. Mutations in the *GLA* gene causing α-galactosidase A deficiency underlie the lysosomal storage disorder Anderson–Fabry disease (OMIM #301500). In this disease, α-galactose-terminated GSLs accumulate in tissues, plasma, and urine. Gb3 and its deacetylated derivative lysoGb3 are the main accumulating GSLs and serve as disease biomarkers [59]. LysoGb3 most probably emerges as a product of acid ceramidase (*N*-acylsphingosine deacylase, EC 3.5.1.23), encoded by the *ASAH1* gene, which can use Gb3 as a substrate when it is available in excess [60]. The GSL deposits also contain galabiosylceramide, the P1 antigen, and the B histo-blood group antigen [61]. The deposits are found in many tissues, particularly in the heart, kidney, peripheral nerves, eye, brain, skin, gastrointestinal tract, and auditory system [62]. In the kidney, GSLs accumulate in podocytes, glomerular endothelial, mesangial, and interstitial and distal tubular cells [63]. Interestingly, in a symptomatic mouse model of Fabry disease (generated by *GLA* gene knockout and human *A4GALT* overexpression), the highest accumulation of Gb3 was found in the spleen and liver, which do not display any abnormalities in the mouse model or patients [64]. Anderson–Fabry disease includes neurological, cardiac, renal, ocular, gastrointestinal, dermatological, and auditory symptoms, of which cardiac and renal dysfunction are the leading causes of death [65]. Approved treatment options involve enzyme replacement (agalsidase alfa and beta) and chaperone therapy (migalastat) [66]. Other agents for enzyme replacement therapy and for substrate reduction approach inhibiting glucosylceramide synthase (ceramide glucosyltransferase, *UGCG*) are also extensively studied. In addition, gene and messenger RNA (mRNA)-based therapies that focus on *GLA* are considered as a viable option. However, clustered regularly interspaced short palindromic repeats (CRISPR)/Cas9-mediated suppression of *A4GALT* seems to emerge as a novel substrate deprivation strategy [67, 68], and perhaps in the future small-molecule inhibitors of Gb3/CD77 synthase could be developed.

## Gb3/CD77 synthase products as receptors for bacterial toxins and adhesins

Galα1 → 4Gal structures produced by Gb3/CD77 synthase are recognized by bacterial adhesins and toxins (reviewed in Refs. [45, 69, 70]). Gb3 is a crucial receptor for Shiga toxins (also known as “verotoxins” or “Shiga-like toxins”), which are major virulence factors released by Shiga toxin-producing *Escherichia coli* (STEC) and *Shigella dysenteriae* of serotype 1 [71]. Shiga toxins are composed of the catalytically active A subunit and a pentamer of B subunits that recognize cellular receptors; toxins with such structure are collectively referred to as AB_5_ class [72]. STEC infections can cause hemorrhagic colitis, which can deteriorate into hemolytic uremic syndrome, a life-threatening complication characterized by thrombocytopenia, anemia, and acute kidney failure [73]. Recently, it was shown that glycoproteins carrying P1 glycotopes can also serve as functional receptors for type 1 Shiga toxins [13, 74]. Galabiosylceramide and the GSL P1 antigen, as well as globotetraosylceramide (Gb4), showed Shiga toxin binding in early reports [75, 76]. However, recent studies corroborated only type 1 Shiga toxin interaction with P1, and its role in toxin internalization still requires elucidation [77].

Staphylococcal enterotoxin B (SEB) from *Staphylococcus aureus* is a major toxin responsible for food poisoning and toxic shock syndrome. It recognizes terminal Galα1 → 4Gal structures, preferring galabiosylceramide [78]. PapG adhesins, which are expressed at the top of uropathogenic *E. coli* (UPEC) P-fimbriae, bind both terminal and internal Galα1 → 4Gal structures [79, 80]. Similarly, SadP adhesin from *Streptococcus suis*, a zoonotic pathogen, which causes meningitis and septicemia, recognizes terminal and internal Galα1 → 4Gal structures present on GSLs and glycoproteins (P1 glycotopes carried by pigeon egg white ovomucoid) [81].

LecA lectin (also known as “PA-I” or “PA-IL”) from *Pseudomonas aeruginosa*, an opportunistic pathogen causing nosocomial infections, binds α-d-galactosylated glycans, including Gb3 and the P1 antigen, with a preference for Gb3 [82, 83]. A LecA homolog, PIIA lectin from *Photorhabdus luminescens*, which lives symbiotically in insect-infecting *Heterorhabditis* nematodes, also recognizes α-d-galactosylated glycans [84].

## The role of Gb3/CD77 synthase and its products in cancer progression

Gb3 overexpression was discovered in Burkitt’s lymphomas, and Gb3 was designated as Burkitt’s lymphoma antigen [85, 86]. Later, increased Gb3 level was reported in colorectal cancer [87–89]. In a study by Kovbasnjuk and coworkers [87], Gb3 expression was linked to a metastatic and invasive phenotype; however, subsequent reports did not corroborate the correlation between Gb3 level and metastasis. Moreover, strong Gb3 expression was found in gastric adenocarcinoma in most tissues of intestinal and diffuse type [90]. Gb3 was also reported as one of the major GSLs in scirrhous gastric carcinoma tissues [91]. Increased Gb3 levels were detected in pancreatic cancer [89, 92]. Breast tumors of distinct clinicopathological types and their lymph node metastases showed increased Gb3 expression, predominantly in epithelial cancer cells [93–95]. Furthermore, different histological subtypes of epithelial ovarian cancer display Gb3 overexpression [96, 97]. Taking advantage of both Gb3 characteristics, as a tumor-associated antigen and Shiga toxin receptor, several therapeutic strategies are now being developed involving targeting cancer cells with Shiga toxin subunit B conjugated to chemotherapeutic drugs [98].

The P1 antigen has been found in serous ovarian cancer and endometrioid peritoneal cancer tissues, as well as on ovarian cancer cells IGROV-1, where it correlated with migratory potential [99]. Moreover, patients with tubal, peritoneal, and ovarian cancer show a decreased level of plasma-derived naturally circulating anti-P1 IgM antibody, and this observation can be applied in biochemical diagnosis of gynecological tumors [99]. Recently, P1 antigen expression was detected in pancreatic ductal and gastric adenocarcinoma [14, 100]. In the latter study, in addition to the classic pentaosylceramide P1, two novel GSL structures terminating with the P1 glycotope were identified: heptaosylceramide and a branched decaosylceramide carrying two P1 trisaccharide units.

Gb3/CD77 synthase initiates the biosynthesis of globosides (GSLs of the globo-series) and plays a particularly important role in the epithelial-to-mesenchymal transition (EMT) of cancer cells. EMT is often accompanied and even induced by changes in the GSL composition of the plasma membrane [97, 101]. It was found that the transcript level of the *A4GALT* gene (encoding Gb3/CD77 synthase) correlates negatively with mesenchymal characteristics in ovarian cancer. Its CRISPR/Cas9-mediated deletion triggers EMT and chemoresistance of ovarian cancer cells [2]. On the contrary, *A4GALT* knockdown by small interfering RNA (siRNA) decreased the migratory potential of colon cancer epithelial cells [87]. In pancreatic ductal adenocarcinoma cells, Gb3 expression was observed in the mesenchymal-like PaTu-8988T but not in the epithelial-like PaTu-8988S cell line [102].

Enhanced biosynthesis of GSLs originating from glucosylceramide (GlcCer) was demonstrated in many chemoresistant cancer cells [103]. In particular, Gb3 overexpression is associated with chemoresistance of several types of human cancer cells, such as ovarian, breast, and colon cancer [104–106]. Interestingly, the increased Gb3 level may induce radio- and chemoresistance of cancer cells through interactions with Hsp70 and cSrc, respectively, in plasma membrane lipid rafts [104, 107]. Cancer cells often show Hsp70 overexpression and abnormal localization in the plasma membrane, which contributes to their radioresistance [108]. It was also shown that interaction with Gb3 enables cancer-specific membrane localization of Hsp70 in colon and pancreatic cancers, as well as Burkitt’s lymphoma cells [107, 109, 110]. On the other hand, association of Gb3 and cSrc kinase in lipid rafts underlies the overexpression of p53 R273H mutant and chemoresistance of colon cancer cells [106]. Furthermore, interactions of Gb3 with cSrc kinase most probably cause overexpression of MDR1 (P-glycoprotein), triggering multidrug resistance in ovarian cancer cells [104]. Gb3 was also suggested to activate the Akt and ERK1/2 signaling pathways leading to MDR1 overexpression and resistance of breast cancer cells [105].

## Conclusions and future perspectives

Human Gb3/CD77 synthase continues to astound and shift paradigms. Once considered an unremarkable single-product enzyme, it has emerged as a multifunctional glycosyltransferase, capable of synthesizing several different structures on GSLs and glycoproteins. While recent studies have significantly expanded our understanding of its activity, other yet-undiscovered glycoconjugates modified by Gb3/CD77 synthase may exist. Preliminary results obtained by Bereznicka and coworkers suggest that a recombinant soluble catalytic domain of Gb3/CD77 synthase can attach galactose residues to Galβ1 → 3GalNAc-terminated O-glycans, forming Galα1 → 4Galβ1 → 3GalNAc structures (Bereznicka et al. unpublished data). However, production of these structures in vivo is a matter of controversy, since O-glycans are readily capped by sialyltransferases with terminal Neu5Ac residues, thus presumably preventing elongation by Gb3/CD77 synthase.

The question that then arises is: why does human Gb3/CD77 synthase show a preference for GSL over glycoprotein acceptors (we estimate it forms 95% of Galα1 → 4Gal structures on GSLs)? The ABO histo-blood group system transferases, which are similarly capable of using both GSLs and glycoproteins as acceptors, synthesize the A and B blood group antigens primarily on glycoproteins (80% of ABH determinants on RBCs) and to a lesser extent on GSLs (20%) [111]. However, a recombinant soluble catalytic domain of Gb3/CD77 synthase efficiently galactosylates purified N-glycans and N-glycoproteins [13]. In the Golgi apparatus, glycosyltransferases are known to form homo- and heterooligomers, which significantly impacts their activity. Such multienzyme complexes are thought to act as assembly lines whereby the products of one reaction move along to become acceptors of the next reaction in the biosynthetic pathway [112, 113]. We presume that the low level of P1 glycotope biosynthesis on cellular glycoproteins may originate from preferential contribution of Gb3/CD77 synthase to multienzyme complexes responsible for GSL production in the Golgi. Recently, Mikolajczyk and coworkers provided evidence for the formation of Gb3/CD77 synthase homodimers as well as heterodimers with β1,4-galactosyltransferase 1 (*B4GALT1*) and β1,4-galactosyltransferase 5 (*B4GALT5*) [114]. Interestingly, Gb3/CD77 synthase showed preference for interactions with β1,4-galactosyltransferase 5, which is a GSL-specific enzyme, in contrast to glycoprotein-specific β1,4-galactosyltransferase 1.

The acceptor promiscuity of human Gb3/CD77 synthase, making it capable of synthesizing both Galα1 → 4Gal and Galα1 → 4GalNAc terminal structures, is a unique property among glycosyltransferases. As the crystal structure of Gb3/CD77 synthase remains to be solved, we can only speculate how p.Q211E substitution enables the enzyme to produce the Galα1 → 4GalNAc moiety of the NOR antigen. We suppose that this modification deepens the acceptor binding site in the enzyme catalytic center, enabling GalNAc-terminated GSL acceptors to fit in. Preliminary analysis of a Gb3/CD77 synthase model predicted by AlphaFold supports this hypothesis (Sobala et al., unpublished data).

The abundance of Gb3/CD77 synthase products in human tissues underscores their importance in bacterial infections, of which STEC seem to pose the greatest threat. The estimated number of acute illnesses worldwide caused by STEC infections is 2.8 million annually, and the disease is particularly dangerous for children and elderly persons [115]. Furthermore, 5–15% of patients develop severe sequelae called hemolytic uremic syndrome, which can be fatal [116].

The current state of knowledge reveals only a glimpse of the potential role that Gb3/CD77 synthase and its products play in the metastasis of cancer cells and their chemoresistance. Both TM9SF2 and GOLPH3, which are crucial for Gb3/CD77 synthase localization in the Golgi and hence influence its activity, were identified as oncoproteins. TM9SF2 has been implicated in the progression of colorectal and pancreatic cancer [117, 118]. GOLPH3 contributes to the tumorigenesis of lung, breast, colorectal, prostate, ovarian, gastric, and hepatocellular cancer, as well as melanoma and glioma, through controlling Golgi-to-plasma membrane transport and DNA damage response [119]. We hypothesize that GSL products of Gb3/CD77 synthase play significant roles in oncogenic axes mediated by GOLPH3 and TM9SF2, potentially opening up a new chapter in the complex and intriguing story of this glycosyltransferase.

## Data Availability

Not applicable.

## Abbreviations

CD77: Cluster of differentiation 77
CRISPR: Clustered regularly interspaced short palindromic repeats
CTH: Ceramide trihexoside
EGR1: Early growth response protein
EMT: Epithelial-to-mesenchymal transition
Ga2: Galabiosylceramide
Gb3: Globotriaosylceramide
Gb4: Globotetraosylceramide
GlcCer: Glucosylceramide
GOLPH3: Golgi phosphoprotein3
GSL: Glycosphingolipid
ISBT: International Society of Blood Transfusion
LAPTM4A: Lysosome-associated protein transmembrane 4α
mRNA: Messenger RNA
ORF: Open reading frame
RBC: Red blood cel
RUNX1: Runt-related transcription factor1
SEB: Staphylococcal enterotoxin B
siRNA: Small interfering RNA
SNFG: Symbol Nomenclature for Glycans
SNP: Single-nucleotide polymorphism
STEC: Shiga toxin-producing *Escherichia coli*
TGN: *Trans*-Golgi network
TM9SF2: Transmembrane 9 superfamily 2 protein
UPEC: Uropathogenic *Escherichia coli*
